# Link between unmet need and economic status in Bangladesh: gap in urban and rural areas

**DOI:** 10.1186/s12905-022-01752-8

**Published:** 2022-05-14

**Authors:** Zillur Rahman Shabuz, M. Ershadul Haque, Md. Kawsarul Islam, Wasimul Bari

**Affiliations:** 1 Department of Statistics, University of Dhaka, Dhaka, Bangladesh; 2 Department of Mathematics and Statistics, Bangladesh University of Textiles, Dhaka, Bangladesh

**Keywords:** Unmet need, Wealth index, Place of residence, BDHS, Logistic regression

## Abstract

**Background:**

Unmet need for family planning (FP) is a core concept in designing FP programmes and reduction of unmet need for FP can improve reproductive and maternal health services. Bangladesh is still away from achieving the target regarding unmet need for FP. This study aimed to explore the composite effect of economic status and place of residence on unmet need for FP among currently married women of reproductive age in Bangladesh after controlling the effect of other selected covariates.

**Methods:**

The study used the data extracted from the Bangladesh Demographic and Health Survey (BDHS) 2017–2018, which is a nationally representative survey implemented using a stratified two-stage cluster sample design. A total of 13,031 currently married women of reproductive age were included in the final analysis. Binary logistic regression model has been employed to identify the factors influencing the unmet need for FP. Model-I investigated the effect of composite variable place-wealth on unmet need for FP and Model-II examined the effect of place-wealth on unmet need for FP after adjusting for the effect of other selected covariates. The Odds Ratios with p-values were reported to identify significant covariates.

**Results:**

The rate of unmet need for FP was 15.48%. The composite factor of economic status and place of residence had significant influence on unmet need for FP in both models. Generally, rural women were significantly more likely to have unmet need for FP than their urban counterparts. In particular, women from rural areas and belong to rich families had the highest likelihoods of unmet need for FP. The other selected covariates also had significant influence on unmet need for FP.

**Conclusion:**

This study shows that rural women had higher odds of unmet need for FP than urban women. The healthcare providers and stakeholders should take necessary actions to motivate women to use contraceptive specially the women who are residing in the rural areas.

## Introduction

Pregnancy related complicacies such as maternal morbidity and mortality can be reduced to a great extent by FP programme for the women of reproductive age [1]. One of the key indicators of national FP programmes is the unmet need for contraceptive [2]. The condition of wanting to avoid or postpone childbearing but not using any method of contraception is referred to as unmet need for FP. It has been a core concept in world population for more than three decades [3–5]. Using three KAP (knowledge, attitudes and practices regarding FP) surveys in Taiwan, Freedman et al. first identified a group of women who wish to stop childbearing but were not using any method of contraceptive [6]. Freedman and Coombs used survey data from several countries to estimate the size of this group [7]. In 1990s, unmet need for FP decreased from 19 to 17% worldwide. But due to population growth, the total number of women having unmet need for FP has remained nearly constant [8]. The proportion of women having unmet need for FP is higher in the developing countries than developed countries [5, 9].

The level of current use of modern contraceptive is a useful measure of success in a FP programme and fruitful in reducing fertility attributable to contraception [5]. In 2015, worldwide 64% of married or in-union women of reproductive age were using traditional or modern method of contraceptive. However, contraceptive use was much lower in the least developed countries (40%) and particularly it was low in Africa (33%) [10]. The reasons for low contraceptive prevalence rate were fear of side effects, health risks, lack of awareness and limited access to the source of supply of contraceptive products [11]. Among the other major geographic areas, contraceptive use was much higher in 2015, ranging from 59% in Oceania to 75% in Northern America [10]. The data from 52 developing countries where Demographic and Health Surveys (DHSs) were conducted between 2005 and 2014 reveal that the proportion of married women of reproductive age had unmet need for FP (either modern or traditional) ranges from 8 to 38% [11].

Information about unmet need for FP helps health professionals, policymakers and funders to estimate the increased demand for FP in developing countries for decades [11]. In 2014, out of the estimated 1.6 billion women of reproductive age in developing countries, 877 million women wanted to avoid childbearing. More than one-fourth of these women (225 million) had unmet need for modern contraceptive [12]. In the developing world, an estimated 74 million unintended pregnancies happened each year and that resulted an estimated 36 million abortions and 8 million (estimated) miscarriages. More than half of these abortions (an estimated 20 million) were unsafe [12] and unsafe abortion can result serious injury, even death [11].

The Government of Bangladesh (GoB) has signed the Millennium Development Goal (MDG) in 2000. The fifth goal of MDGs was to reduce the unmet need for FP to at most 7.6% by 2015 [13]. The rate of unmet need for FP was 15.3% in Bangladesh at the time of singing MDGs [14]. Among the married women of reproductive age in Bangladesh, 62% were using any method of contraceptive in 2017–2018 and 12% of married women had unmet need for FP [15], whereas these figures were 61.2% and 14% respectively in 2011 [16]. Therefore, Bangladesh was far from achieving the MDG goal in 2011 regarding unmet need for FP. In the 2012 international FP summit, the Family Planning 2020 agenda was targeted to get access additional 120 million women on to the use of modern contraceptives from 69 poorest developing countries in the world [17]. The GoB has declared to lower the unmet need for FP in the international FP summit 2012 to at most 7% by 2021. According to NIPORT, the rate of unmet need for FP was reduced from 15.3% to 12.0% during 2000–2014 [18]. Under this circumstance, the GoB has revised the target in 2015 regarding unmet need for FP to reach it to at 10% by 2021 [19].

Several studies have been conducted to measure the proportion and to identify the factors affecting unmet need for FP in Bangladesh. Ferdousi et al. conducted a study in Sreepur upazila under Gazipur district and concluded that 22.4% women had unmet need for FP and the main reason for not using contraceptive was fear of side effects [20]. Islam et al. utilized BDHS 2004 data to identify the factors that influence unmet need for FP in rural and urban areas [21]. They identified that factors of unmet need for FP varied with the place of residence, i.e., rural and urban areas. Similar finding was observed in a recent study carried by Khan et al. [22]. The focus of the Pradhan and Dwivedi's study was to identify the community level covariates in addition to individual and household level covariates affecting unmet need for FP using BDHS 2011 data [23]. Using BDHS 2014 data, Khatun and Mallick concluded that unmet need for FP was found to be higher in the rural areas, among Muslims, among women who were not exposed to media or not member of any NGO, among couples having no sons [24]. A study conducted by Barkat-e-khuda et al. using BDHS 1993–1994 and 1996–1997 datasets identified ever use of FP, husband-wife communication on FP matter, number of living children, and place of residence as the main predictors [25]. However, none of the authors had considered wealth gap among rural and urban residence in studying unmet need for FP.

Most of the studies on unmet need for FP reported that the place of residence and wealth index were important determinants of unmet need [23, 24, 26]. Unmet need for FP is higher in the rural areas than the urban areas in most countries. Some exception was found for most of the countries of sub-Saharan Africa. Wealth index has been found to have an inverse relation with the unmet need for FP, except for some of the least develop countries [27]. To the best of our knowledge, the composite effect of place of residence and wealth on unmet need for FP has yet not been studied by any researcher. In this study, the main goal is to explain whether role of wealth index on unmet need for FP is same both in urban and rural areas of Bangladesh or not. For this purpose, a composite factor has been created using wealth index and place of residence and a link between this composite factor and unmet need for FP has been investigated.

## Data and methods

### Data

The data used in this study was taken from the BDHS 2017–2018, a secondary dataset, which is available in https://dhsprogram.com/data/available-datasets.cfm. It is the latest available DHS data set in Bangladesh consisting of information about unmet need for FP. The BDHS 2017–18 is a nationally representative survey in which the whole country was divided into eight administrative divisions: Barishal, Chattogram, Dhaka, Khulna, Mymensingh, Rajshahi, Rangpur, and Sylhet. Each division was divided into districts, and each district into sub-districts. The survey was based on a two-stage stratified sample of households. In the first stage, 675 clusters were selected with probability proportional to cluster size—250 clusters from the urban areas and 425 from the rural areas. A cluster is a geographic area consisting of an average of 120 households. In the second stage, an average of 30 households from each cluster were selected using systematic sampling procedure. However, the survey was carried out in 672 clusters as one urban cluster and two rural clusters were completely destroyed by floodwater. A total of 20,160 residential households were selected from the 672 clusters and 20,127 ever-married women age 15–49 were successfully interviewed.

### Unmet need for FP

In this study, we have used the revised definition of unmet need for FP proposed by Bradley et al. [28]. Unmet need for FP means the contraceptive need of fecund and currently married or in-union women who are (i) neither pregnant nor postpartum amenorrheic (Postpartum amenorrheic means menstruation not resumed since the delivery of most recent child in the last 2 years.) and wants to space or limit their future births but not currently using any contraceptive, or (ii) whose current pregnancy was mistimed or unwanted, or (iii) postpartum amenorrheic and whose most recent birth in the last 2 years was mistimed or unwanted. Here spacing refers that woman does not want child within the next two years, or are undecided whether to have another child or about the timing of the next child; and limiting means woman does not want more children.

The total unmet need for FP is the sum of unmet need for spacing and limiting. Women who were using any method of contraceptive were considered to have met need for FP [18, 28]. The present study includes women who had either met need or unmet need for FP. This study excludes women who were pregnant and postpartum amenorrheic whose pregnancy was the result of a contraceptive failure, and women who had no need for contraceptive methods, either because they desire a child soon (within the next two years) or because they were menopausal or infecund. Also the data set was filtered for missing values in the selected covariates. Finally, the number of women reduced to 13031 using the criteria mentioned above. Hence the data contain information obtained from 13031 women.

In the standard calculation of unmet need for FP the denominator is all currently married or in union women of reproductive age [28, 29]. The NIPORT used all currently married women of age 25–49 as the denominator [15]. However, in our study we have considered total demand for FP of currently married women of reproductive age (currently married women of reproductive age who had unmet need for FP plus who were using any method of contraceptive) which is the subset of all currently married women of reproductive age. Hence our estimate of rate of unmet need for FP is higher than the figure reported in NIPORT [15].

### Variables

The response variable of interest is unmet need for FP which has two categories: unmet need and met need. Based on the objective of the study we have considered wealth index and place of residence as the main covariates. A number of other available important variables from BDHS 2017–2018 were considered as controlled covariates. The full list of variables along with the categories that were used in our study is given in Table 1.

**Table 1:** Variables and their categories

Variable type	Variable name	Category
Dependent	Unmet need	No, Yes
Covariate	Wealth Index	Not rich, Rich
	Place of residence	Rural, Urban
	Spousal education level	Both spouse have below higher education; Woman has below higher but her spouse has higher education; Woman has higher but her spouse has below higher education; Both spouse have higher education
	Respondent working status	No, Yes
	Sex composition	No child, Only 1 or 2 sons, Only 1 or 2 daughters, 1 son and 1 daughter, 3 or more children
	Migration time	Non-migrant, 6 or more years, 0–5 years
	Visited by FP workers	No, Yes
	Religion	Muslim, Non-Muslim
	Division	Barishal, Chattogram, Dhaka, Khulna, Mymensing, Rajshahi, Rangpur, Sylhet

All of the variables and their categories presented in Table 1 were not readily available in BDHS 2017–2018 data set. The wealth index which is a composite measure of consumer goods a household own, calculated using principal component analysis, was categorized as not rich (lower 66.67 percentile) and rich (upper 33.33 percentile). Spousal education was derived from the respondent’s education level and her husband’s education level and was categorized into four groups given in Table 1. The respondent who always live in her current place of residence was considered as non-migrant; otherwise considered as migrant. Visitors were considered as missing and hence excluded from the analysis. The migrants were categorized into settled migrants (live at the current residence for 6 or more years) and recent migrants (live at the current residence for five or fewer years).

### Methods

We performed univariate analysis (frequency distribution) to see the basic characteristics of the explanatory variables in the sample and bivariate analysis to explore the association between dependent variable and selected explanatory variables by using chi-square test. For calculating the chi-square statistic we considered the weighted observed cell counts and hence the percentages displayed in Table 2 are based on weighted observed counts. The reason for considering the weighted observed counts is to address the non-proportionality of selecting households from the strata within the design of proportional allocation in DHS data. For regression analysis, binary logistic regression model has been employed to find the unadjusted and adjusted effects of covariates on unmet need for FP. Covariates found to have significant association with the unmet need for FP in the bivariate analysis were considered in the regression analysis. For analyzing data, statistical software STATA 14 has been employed. The svyset command has been used in the bivariate and regression analysis to address the stratified two-stage cluster sampling design.

**Table 2:** Percentage distribution of unmet need for FP by selected explanatory variables

Variable	n (%)	Unmet need (%)		*p* value
		No	Yes	
Unmet need		84.52	15.48	
Place-wealth				< 0.001
Urban-not rich	1976 (15.16)	88.15	11.85	
Urban-rich	2864 (21.98)	88.64	11.36	
Rural-Not rich	6791 (52.11)	84.23	15.77	
Rural-rich	1400 (10.74)	76.85	23.15	
Spousal education level				< 0.001
H^−^ W^−^	10,360 (79.50)	84.51	15.49	
H^+^ W^−^	930 (7.14)	83.79	16.21	
H^−^ W^+^	480 (3.68)	75.01	24.99	
H^+^ W^+^	1261 (9.68)	89.32	10.68	
Respondent working status				< 0.001
No	6434 (49.37)	80.51	19.49	
Yes	6597 (50.63)	88.43	11.57	
Sex composition				< 0.001
No child	568 (4.36)	71.76	28.24	
Only 1 or 2 sons	2649 (20.33)	83.64	16.36	
Only 1 or 2 daughters	1998 (15.33)	83.45	16.55	
1 son and 1 daughter	2616 (20.08)	85.77	14.23	
Three or more children	5200 (39.90)	86.16	13.84	
Migration time				< 0.001
Non-migrant	1667 (12.79)	80.19	19.81	
6 or more years	8252 (63.33)	86.12	13.88	
0–5 years	3112 (23.88)	82.58	17.42	
Visited by FP workers				< 0.001
No	9967 (76.49)	82.53	17.47	
Yes	3064 (23.51)	91.06	8.94	
Religion				< 0.001
Muslim	11,661 (89.49)	83.65	16.35	
Non-Muslim	1370 (10.51)	92.34	7.66	
Division				< 0.001
Barishal	1424 (10.93)	81.80	18.20	
Chattogram	1775 (13.62)	76.50	23.50	
Dhaka	1992 (15.29)	84.11	15.89	
Khulna	1717 (13.18)	88.61	11.39	
Mymensing	1409 (10.81)	86.94	13.06	
Rajshahi	1706 (13.09)	87.68	12.32	
Rangpur	1705 (13.08)	90.22	9.78	
Sylhet	1303 (10.00)	80.65	19.35	

(1) The last column presents *p* values of chi-square test statistics, (2) H: Husband, W: Woman, + Higher level of education, − Below higher level of education

#### Chi-square test

Suppose that the data on two categorical variables are displayed in a contingency table and there are $r$ categories of the row variable and $c$ categories of the column variable. The chi-square statistic is then defined as

1 $${\chi }^{2}=\sum_{i=1}^{r}\sum_{j=1}^{c}\frac{({O}_{ij}-{E}_{ij}{)}^{2}}{{E}_{ij}},$$

where ${O}_{ij}$ denotes the observed cell count for $i$ th category of the row variable and $j$ th category of the column variable and ${E}_{ij}$ is its corresponding expected cell count. The quantity in Eq. (1) is a chi-square random variable with $\left(r-1\right)(c-1)$ degrees of freedom. Therefore, the $p$ value for testing the null hypothesis of independence of two attributes can be computed by using the chi-square distribution with $\left(r-1\right)(c-1)$ degrees of freedom and can make decision accordingly.

#### Logistic regression model

Suppose that ${Y}_{i}$ denotes the binary response for the $i$ th individual ($i=1,\dots ,n$). The value ${Y}_{i}=1$ indicates that $i$ th individual has unmet need for FP and ${Y}_{i}=0$ otherwise. The probability of outcome event for the $i$ th individual, $\mathrm{Pr}\left({Y}_{i}=1\right)={\pi }_{i}$, can be modelled using logit link function as

2 $$\mathrm{logit}\left({\pi }_{i}\right)=\mathrm{log}\left[\frac{{\pi }_{i}}{1-{\pi }_{i}}\right]={\beta }_{0}+{\beta }_{1}{x}_{i1}+\dots +{\beta }_{k}{x}_{ik},$$

where ${x}_{ij}$ is the value of $j$ th covariate for $i$ th individual and ${\beta }_{j}$ is the corresponding regression coefficient.

## Results

Table 2 presents the frequency distribution and its corresponding percentage distribution of each category of the covariates as well as the weighted percentage distribution of each explanatory variable categories by the categories of the response variable. This table also presents the $\mathrm{p}$-value of chi-square statistic for measuring the association between each specified explanatory variable and the response variable. In Table 2, percentages in column 2 are un-weighted percentages while the percentages in column 3 and 4 were obtained using the effect of complex multistage survey design.

As mentioned earlier, a total of 13031 women (un-weighted) were considered in this study. The results in Table 2 show that the proportion of unmet need for FP in Bangladesh was 15.48%. Most of the respondents (52.11%) were from rural areas and were not rich while the least (10.74%) were also from rural areas and whose economic status were rich. In most cases (79.50%) both husband and wife had below higher education and in 9.68% cases both spouse had higher education while only in 3.68% cases wife had higher education and her husband had below higher education. Approximately 50% of the women were working at the time of survey. A greater portion of women (39.90%) had three or more children whereas only 4.36% women had no children. Only 12.79% women were non-migrant and about two-third of women (63.33%) were settled migrant. More than three-fourth of women (76.49%) reported that FP workers never visited them in the six months before the survey. Around 90% of women were Muslim and remaining women were non-Muslim. The highest percentage of women were from Dhaka division (15.29%) and lowest from Sylhet division (10.00%) whereas the percentages for Barishal and Mymensing division were very close to Sylhet division.

The chi-square test has been employed to assess whether a specific explanatory variable was significantly associated (unadjusted association) with unmet need for FP. From Table 2 it is observed that all explanatory variables are significantly associated with unmet need for FP at 1% level of significance. Women who live in the rural areas and are rich had the highest (23.15%) unmet need for FP followed by not-rich women in the rural areas and this figure is approximately equal to the overall unmet need for FP of 15.48%. However, as expected women of both rich and not-rich families from the urban areas had low level of unmet need for FP. Husband’s education plays an important role in reducing unmet need for FP. Unmet need for FP was highest (24.99%) if wife has higher education but her husband has below higher education and was lowest (10.68%) if both spouse have higher education. Working women had less unmet need for FP (11.57%) than not working group (19.49%). Unmet need for FP was highest for women who have no children (28.24%) and was lowest for women having 3 or more children (13.48%). One of the probable reasons is that women who have no children were not aware about FP specifically the issue of unmet need for FP for spacing. Non-migrant women had highest (19.81%) unmet need for FP followed by recent migrant women while settled migrant had lowest (13.88%) unmet need for FP. The result clearly indicates that women who are visited by FP workers in the last six months had around 10% reduced unmet need for FP than women who are not visited by FP workers. Muslim women had about 10% higher unmet need for FP than other religious communities. Unmet need for FP was highest (23.50%) in Chattogram division which was more than twice of Rangpur division (9.78%), the lowest unmet need for FP among eight divisions.

In the bivariate analysis, all covariates have found significant association with unmet need for FP (unadjusted association) and hence all covariates were considered into logistic regression analysis to determine the adjusted effects of these covariates on unmet need for FP. The main focus of this study was to investigate the link of wealth on unmet need for FP and their rural and urban gap. Hence a composite factor of wealth index and place of residence (place-wealth) was considered as the exposure variable. The unadjusted effect of the composite variable place-wealth on unmet need for FP was considered in Model-I and its effect after adjusting other covariates was also considered in Model-II. Table 3 presents the results of unadjusted and adjusted effects (Odds Ratios) on unmet need for FP along with p-values.

**Table 3:** Odds ratio and *p* value of logistic regression models for the determinants of unmet need for FP

Variable	Logistic model-I (unadjusted)		Logistic model-II (adjusted)	
	Odds ratio	*p* value	Odds ratio	*p* value
Intercept	0.134	< 0.001	0.546	< 0.001
Place-Wealth				
Urban-not rich (Ref)				
Urban-rich	0.953	0.638	0.808	0.053
Rural-not rich	1.392	< 0.001	1.581	< 0.001
Rural-rich	2.239	< 0.001	2.222	< 0.001
Spousal education level				
H^−^ W^−^ (Ref)				
H^+^ W^−^			0.927	0.519
H^−^ W^+^			1.411	0.015
H^+^ W^+^			0.545	< 0.001
Respondent working status				
No (Ref)				
Yes			0.652	< 0.001
Sex composition				
No child (Ref)				
Only 1 or 2 sons			0.595	< 0.001
Only 1 or 2 daughters			0.606	< 0.001
1 son and 1 daughter			0.527	< 0.001
Three or more children			0.428	< 0.001
Migration time				
Non-migrant (Ref)				
6 or more years			0.729	< 0.001
0–5 years			0.751	0.003
Visited by FP workers				
No (Ref)				
Yes			0.477	< 0.001
Religion				
Muslim (Ref)				
Non-Muslim			0.451	< 0.001
Division				
Barishal (Ref)				
Chattogram			1.418	0.004
Dhaka			0.958	0.751
Khulna			0.663	0.001
Mymensingh			0.748	0.046
Rajshahi			0.637	0.003
Rangpur			0.589	< 0.001
Sylhet			1.056	0.686

H: Husband, W: Woman, + Higher level of education, − Below higher level of education

The unadjusted model shows that the composite variable place-wealth had significant effect on unmet need for FP. This model depicts that women who live in rural areas and whose economic status is not-rich were 1.392 times as likely to have unmet need for FP, whereas women who are rich and live in rural areas were more than twice as likely to have unmet need for FP as women who live in urban areas and are not rich.

In the adjusted model, all the selected covariates had significant effect on unmet need for FP. The results show that women who live in rural areas and rich were 2.22 times as likely to have unmet need for FP as women who live in urban areas having not rich economic status. However, the likelihood of unmet need for FP was around 20% lower among women who live in urban areas and they are rich. It is interesting to note that unmet need for FP behaved differently for rich women in rural and urban areas. Spouses both having higher education were approximately 45% less likely to have unmet need for FP than spouses both having below higher education whereas if women have higher education but their husbands have below higher education then unmet need for FP increased by 41%. Working women had 35% lower odds of having unmet need for FP than those who are not working at the time of the survey. Families having one or more children were less likely to have unmet need for FP than those families having no children. Settled migration reduced unmet need for FP by 27% whereas recent migration reduced it by 25% than non-migrant. The likelihood of unmet need for FP was 52% lower among women who are visited by FP workers in the last six months before the survey than those who are never visited by FP workers. Non-Muslim women had 55% lower odds of having unmet need for FP than Muslim women. Women from Chattogram division were 1.418 times more likely to have unmet need for FP than women from Barishal division. While women who live in Khulna, Mymensingh, Rajshahi and Rangpur divisions had 33.3%, 25.2%, 36.3% and 41.1% lower odds of having unmet need for FP respectively compared to women who live in Barishal division.

## Discussion

There were several studies that explore the proportion and determinants of unmet need for FP in Bangladesh [21, 23, 24]. This study examined the effect of wealth index on unmet need for FP in Bangladesh and how this effect varies between rural and urban areas. In determining the required adjusted effect few selected covariates were used as control covariates. The data used in this study have been extracted from the BDHS 2017–2018 data. We have considered the strict definition of infecundity in defining unmet need for FP and only the number of currently married women of reproductive age who have demand for FP were used in the denominator to calculate the proportion of unmet need for FP. However, the NIPORT used all currently married women of reproductive age as a denomination when calculating the percentage of unmet need for FP [15].

The proportion of unmet need for FP among currently married women of reproductive age was 15.48% in Bangladesh. This proportion was approximately equal to the figure of Nigeria (16.1%) and was less than the figure of Malawi (21.0%) [26, 30]. However, both Nigeria and Malawi's studies considered all sexually active women (married or unmarried) of reproductive age. It is expected that figures of those two studies should be higher than the figure of Bangladesh as young unmarried people face great barriers to access FP services and hence may have higher unmet need for FP than the married people [21, 23, 27].

Using BDHS 2014 data, Khatun and Mallick found that women from rich families had significantly higher odds of having unmet need for FP than women from poor families (OR = 1.25, CI: 1.08–1.46) [24]. In other studies, wealth index had no significant effect on unmet need for FP [23]. However, studies in other countries reported that poor women were more likely to have unmet need for FP [26, 30]. Huda et al. extensively reviewed literature from ten articles on contraceptive use in Bangladesh and observed that rural women had significantly higher odds of having unmet need for FP than urban women [31]. A similar finding was found in Ethiopia [32]. In this study, using the composite factor place-wealth we have found that women from rural areas and rich families had the highest likelihoods of having unmet need for FP (OR = 2.222, *p* < 0.001) compared to women of urban areas and not rich families. However, women of urban areas and rich families reduced unmet need for FP.

The present study demonstrated that higher educated spouses were less likely to have unmet need for FP than the below higher educated spouses. Note that an earlier study in Nigeria [26] had also found a similar effect of education of women on unmet need for FP. However, Islam et al. reported that in Bangladesh higher educated women had more odds of having unmet for FP to space compared to illiterate women [21]. In Kenya and Eastern Sudan, educational attainment (at least secondary level) of the respondent significantly reduce the unmet need for FP [33, 34].

As expected, working married women had less odds of unmet need for FP than those women who are not working at the time of the survey. This is consistent with studies from Ghana and Malawi [30, 35] and a study in Bangladesh [21]. Working women may have easy access to quality health services and have better autonomy in using contraceptive [30]. However, a contrary findings was found in rural areas of Burkina Faso [5].

In a study in the rural areas of Burkina Faso, it is observed that with living children ($\ge 1$) were much more likely (more than five times) to have unmet need for modern contraceptive than women with no living children [5]. But our study discovered that women with more living children were less likely to have unmet need for FP than women with no living children and is consistent with another Bangladeshi study [24] in which unmet need were likely to reduce in families having more children.

Our findings provide evidence that migrant women were less likely to have unmet need for FP than non-migrant women in Bangladesh. This is consistent with studies in Ethiopia and Cotonou of Benin Republic [36, 37]. Migration can delay childbearing due to time taken to adjust at the new environment [38]. If migration is particularly to search for opportunity, migration provides easy access and better exposure to contraceptive methods [36].

As expected, the likelihood of unmet need for FP was less for the women who are visited by FP workers in the last six months of the survey that corroborates with the figure obtained in Ethiopia and rural areas of Burkina Faso [5, 32]. However, study in Burkina Faso considered whether respondents recently visited a health facility with or without discussing FP issues. Discussion with FP workers improve the knowledge and hence they are eagerly using FP methods [5]. Women whose movement were monitored or cannot discuss FP services were more likely to have unmet need for FP [26].

Consistent with studies from Bangladesh, Nepal and Nigeria [21, 26, 39], in this study it was found that women form Muslim families were more likely to have unmet need for FP compared to non-Muslim women. Religious beliefs reduce the use of contraceptive and hence increase the fertility. In a study among young Muslim couples in Iran revealed that participants having strong religious beliefs desire more children [40]. Muslim women who live in the Northern part of Nigeria usually desire larger families and hence have less demand of contraceptive [26].

The likelihood of having unmet need for FP varies across geographical region and that agree with the results of two studies in Bangladesh [23, 24] and a study in Nigeria [26]. However, unmet need for FP in Sylhet division was significantly higher than that in Barishal division in previous two studies which is not significantly higher in our study.

## Conclusion

The study shows that the role of wealth index on unmet need vary between rural and urban areas of Bangladesh. It was observed that women from rural areas generally had higher likelihoods of unmet need for FP than women from any economic group of urban areas; the unmet need for FP was highest among women of rural areas belonging to rich family. However, the odds of unmet need of FP was lowest among women from urban areas with rich economic status. Beside significant effect of composite factor of wealth index and place of residence on unmet need for FP; spousal education level, working status of women, sex composition of children, migration time, FP workers visited to household, religion and geographical location were the determinants of unmet need for FP in Bangladesh. In order to achieve the target regarding unmet need for FP, healthcare providers and stakeholders should address effective FP programme to enhance accessibility and availability of FP methods by increasing campaign and selective home visits specially in the rural areas.

## Data Availability

The data is available in https://dhsprogram.com/data/available-datasets.cfm.

## Abbreviations

BDHS: Bangladesh demographic and health survey
DHS: Demographic and health survey
GoB: Government of Bangladesh
FP: Family planning
MDG: Millennium development goal
